# Supplementary material for: Screening of Relevant Metabolism-Disrupting Chemicals on Pancreatic β-Cells: Evaluation of Murine and Human In Vitro Models
Source: Int J Mol Sci. 2022 Apr 10;23(8):4182. doi: 10.3390/ijms23084182 (PMC9025712; doi:10.3390/ijms23084182)
Supplement: Supplementary file 1 [file ijms-23-04182-s001.zip › ijms-1675010-supplementary.pdf]

SUPPLEMENTARY MATERIAL

Screening of Relevant Metabolism-Disrupting Chemicals on Pancreatic β-Cells: Evaluation of Murine and Human In Vitro Models

Ruba Al-Abdulla [1], Hilda Ferrero [1,2], Sergi Soriano [1,3], Talía Boronat-Belda [1] and Paloma Alonso-Magdalena [1,2,*]

[1] Instituto de Investigación, Desarrollo e Innovación en Biotecnología Sanitaria de Elche (IDiBE),
Universidad Miguel Hernández, 03202 Elche, Spain; ral@umh.es (R.A.-A.);
hferrero@umh.es (H.F.);
sergi.soriano@ua.es (S.S.); tboronat@umh.es (T.B.-B.)

[2] Centro de Investigación Biomédica en Red de Diabetes y Enfermedades Metabólicas Asociadas
(CIBERDEM), 28029 Madrid, Spain

[3] Departamento de Fisiología, Genética y Microbiología, Universidad de Alicante, 03690 Alicante, Spain

* Correspondence: palonso@umh.es. Paloma Alonso-Magdalena, Instituto de Investigación, Desarrollo e Innovación en Biotecnología Sanitaria de Elche (IDiBE), Universidad Miguel Hernández de Elche, 03202-Elche, Spain.

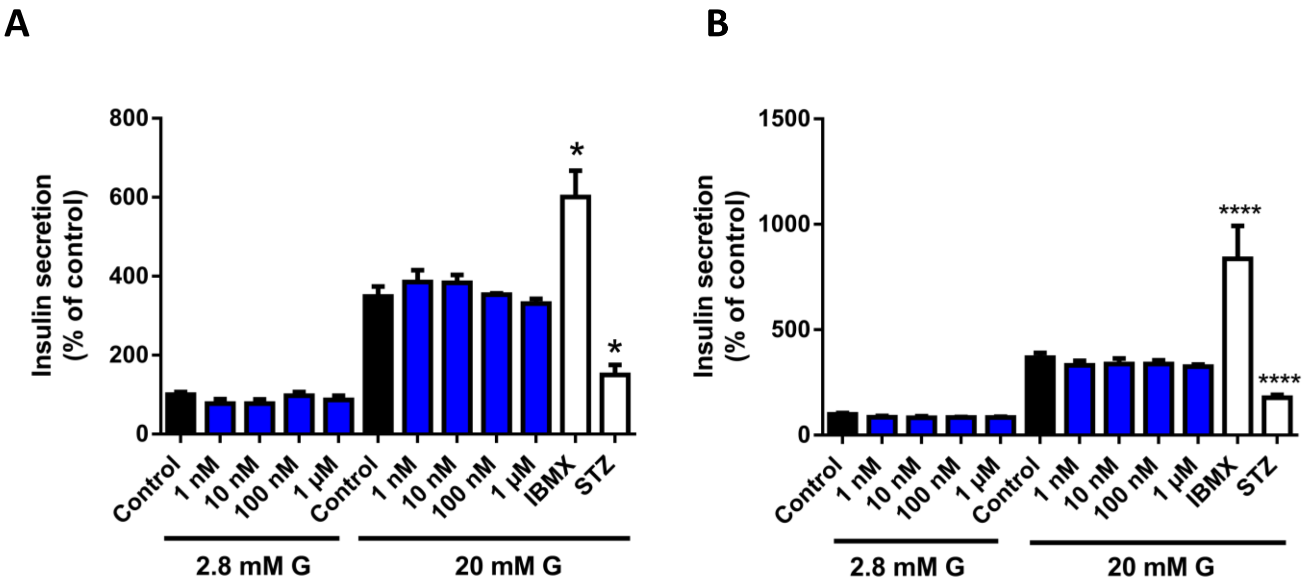

Supplemental Figure S1. Glucose-stimulated insulin secretion (GSIS) in EndoC-βH1 cells treated with BPA (1 nM–1 μM) for 24 h (A) or 48 h (B). n = two independent experiments (A), n = three independent experiments (B). * vs. Control 20 mM G; *p < 0.05, ****p < 0.0001, one-way ANOVA followed by Dunnet's post hoc test. All data are expressed as mean ±SEM.

A

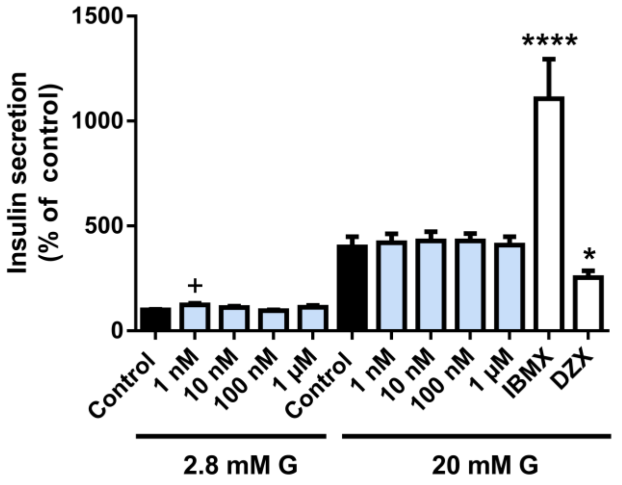

B

24 h	Resazurin	Neutral red	CFDA-AM
Control	100.0 ± 0.5	100.0 ± 1.6	100.0 ± 2.3
BPF 1 nM	98.4 ± 0.6	102.3 ± 1.9	98.4 ± 2.1
BPF 10 nM	96.1 ± 1.0 *	104.6 ± 1.9	94.9 ± 2.1
BPF 100 nM	94.0 ± 1.3 ***	107.1 ± 2.0	90.4 ± 2.3 **
BPF 1 μM	91.2 ± 1.2 ****	109.5 ± 3.0 **	92.5 ± 2.5 *
48 h	Resazurin	Neutral red	CFDA-AM
Control	100.0 ± 0.5	100.0 ± 1.7	100.0 ± 1.6
BPF 1 nM	96.2 ± 0.8	103.1 ± 1.3	95.5 ± 1.8
BPF 10 nM	93.2 ± 0.4 ***	104.2 ± 2.1	99.8 ± 2.0
BPF 100 nM	87.9 ± 1.2 ****	106.7 ± 1.6 *	94.0 ± 1.6
BPF 1 μM	90.0 ± 1.2 ****	106.0 ± 0.9 *	94.2 ± 2.6
72 h	Resazurin	Neutral red	CFDA-AM
Control	100.0 ± 0.7	100.0 ± 1.2	100.0 ± 1.6
BPF 1 nM	99.8 ± 0.7	106.0 ± 1.6 *	94.7 ± 2.9
BPF 10 nM	98.8 ± 1.2	108.5 ± 1.8 ***	98.2 ± 3.1
BPF 100 nM	96.6 ± 1.0	106.2 ± 1.7 *	94.6 ± 2.5
BPF 1 μM	94.5 ± 1.2 ***	105.4 ± 1.1 *	96.3 ± 2.2

Supplemental Figure S2. (A) GSIS in EndoC-βH1 cells treated with BPF (1 nM–1 μM) for 72 h. n = three independent experiments. + vs. Control 2.8 mM G and * vs. Control 20 mM G; +p < 0.05, *p < 0.05, ****p < 0.0001, one-way ANOVA followed by Dunnet's post hoc test. (B) Viability of EndoC-βH1 cells treated for 72 h with different BPF concentrations (1 nM-1 μM) as evaluated by RZ, NR and CFDA-AM assays. n = four independent experiments. * vs. Control; *p < 0.05, **p < 0.01, ***p < 0.001 and

****p < 0.0001, one-way ANOVA followed by Dunnet's post hoc test or Kruskal-Wallis followed by Dunn's post hoc test. All data are expressed as mean ± SEM.

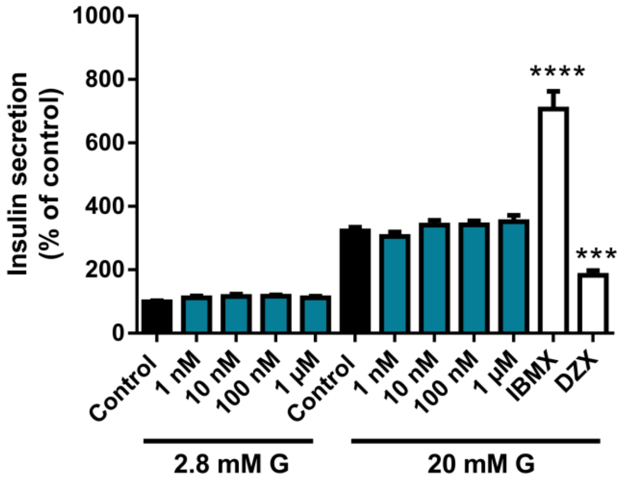

Supplemental Figure S3. GSIS in EndoC-βH1 cells treated with DEHP (1 nM–1 µM) for 72 h. n = six independent experiments. * vs. Control 20 mM G; ***p < 0.001, ****p < 0.0001, one-way ANOVA followed by Dunnet's post hoc test. All data are expressed as mean ± SEM.

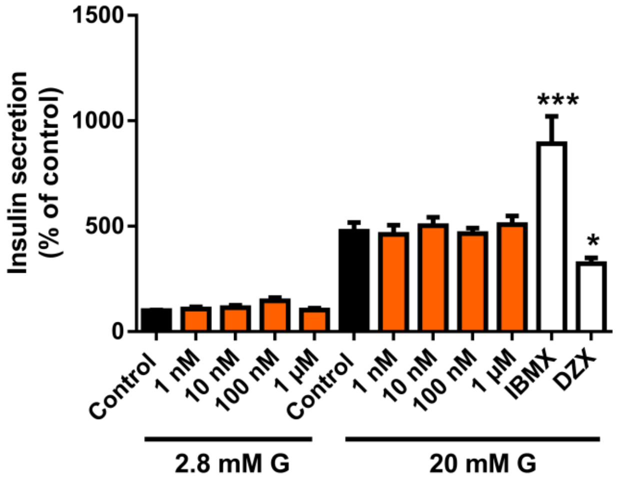

Supplemental Figure S4. GSIS in EndoC-βH1 cells treated with DDE (1 nM–1 µM) for 72 h. n = three independent experiments. * vs. Control 20 mM G; *p < 0.05, ***p < 0.001, ANOVA one way followed by Dunnet's post hoc test. All data are expressed as mean ± SEM.

MIN6							
**48 h**	**Resazurin**	**Neutral red**	**CFDA-AM**	**48 h**	**Resazurin**	**Neutral red**	**CFDA-AM**
**Control**	100.0 ± 0.9	100.0 ± 0.9	100.0 ± 0.8	**Control**	100.0 ± 1.9	100.0 ± 0.8	100.0 ± 0.7
**BPA 100 pM**	93.5 ± 1.4 *	99.9 ± 1.1	96.0 ± 1.0	**BPS 100 pM**	90.6 ± 2.8 *	93.3 ± 1.5 ***	96.5 ± 1.7
**BPA 1 nM**	86.4 ± 2.3 ****	100.5 ± 1.3	90.2 ± 1.6 ****	**BPS 1 nM**	89.0 ± 3.3 *	96.8 ± 1.3	96.6 ± 1.2
**BPA 10 nM**	87.7 ± 1.6 ****	93.3 ± 1.8	92.4 ± 1.2 ***	**BPS 10 nM**	89.2 ± 3.1 *	96.3 ± 1.0	95.1 ± 1.3 *
**BPA 100 nM**	89.7 ± 1.5 ***	100.7 ± 1.3	94.4 ± 1.4 *	**BPS 100 nM**	89.6 ± 2.9 *	97.5 ± 1.1	94.9 ± 1.2 *
**BPA 1 µM**	85.6 ± 2.2 ****	85.1 ± 3.2 *	88.1 ± 1.2 ****	**BPS 1 µM**	87.0 ± 2.6 **	96.6 ± 1.0	92.9 ± 1.1 ***
**BPA 10 µM**	87.4 ± 3.4 **	100.1 ± 1.6	92.0 ± 1.5 ****	**BPS 10 µM**	84.1 ± 2.3 ****	89.9 ± 1.4 ****	93.0 ± 1.2 ***
**72 h**	**Resazurin**	**Neutral red**	**CFDA-AM**	**72 h**	**Resazurin**	**Neutral red**	**CFDA-AM**
**Control**	100.0 ± 1.2	100.0 ± 1.2	100.0 ± 0.7	**Control**	100.0 ± 2.0	100.0 ± 0.7	100.0 ± 1.1
**BPA 100 pM**	89.3 ± 2.8 *	92.4 ± 3.7	94.4 ± 1.6 *	**BPS 100 pM**	97.3 ± 2.3	100.5 ± 0.9	98.1 ± 1.2
**BPA 1 nM**	94.1 ± 2.0	103.9 ± 2.0	98.2 ± 1.2	**BPS 1 nM**	92.6 ± 2.3	101.3 ± 0.9	94.6 ± 1.3*
**BPA 10 nM**	78.2 ± 1.9 ****	85.3 ± 2.3 **	88.4 ± 1.2 ****	**BPS 10 nM**	90.9 ± 2.4 *	99.9 ± 1.1	94.3 ± 1.4**
**BPA 100 nM**	103.8 ± 2.0	116.0 ± 2.5 **	102.3 ± 0.9	**BPS 100 nM**	92.2 ± 2.1	102.9 ± 1.0	94.8 ± 1.3*
**BPA 1 µM**	96.2 ± 2.7	107.5 ± 3.6	96.3 ± 1.5	**BPS 1 µM**	94.3 ± 2.1	102.5 ± 1.1	95.2 ± 1.2*
**BPA 10 µM**	82.8 ± 3.1 ****	83.9 ± 3.5 **	88.9 ± 1.8 ****	**BPS 10 µM**	93.7 ± 2.1	99.9 ± 1.7	95.2 ± 1.3*
**48 h**	**Resazurin**	**Neutral red**	**CFDA-AM**	**48 h**	**Resazurin**	**Neutral red**	**CFDA-AM**
**Control**	100.0 ± 1.1	100.0 ± 1.0	100.0 ± 0.9	**Control**	100.0 ± 3.0	100.0 ± 0.7	100.0 ± 4.2
**PFOS 100 pM**	91.9 ± 1.9 **	93.4 ± 1.9 *	95.6 ± 1.5 *	**$CdCl_2$ 100 pM**	92.6 ± 1.6	87.1 ± 3.8	91.5 ± 3.5
**PFOS 1 nM**	94.4 ± 1.8 *	93.5 ± 2.5 **	99.7 ± 2.0	**$CdCl_2$ 1 nM**	83.3 ± 3.5 **	71.1 ± 2.1 ****	86.7 ± 2.7
**PFOS 10 nM**	89.2 ± 1.2 ****	83.9 ± 2.1 ****	94.5 ± 0.9 **	**$CdCl_2$ 10 nM**	83.9 ± 3.2 *	83.3 ± 3.2	86.1 ± 3.0
**PFOS 100 nM**	92.9 ± 1.5 **	96.8 ± 1.9	97.4 ± 1.0	**$CdCl_2$ 100 nM**	80.2 ± 2.5 **	72.1 ± 3.3 ***	78.2 ± 5.0 *
**PFOS 1 µM**	83.7 ± 2.5 ****	82.7 ± 1.7 ****	86.9 ± 1.8 ****	**$CdCl_2$ 1 µM**	63.4 ± 2.4 ****	56.2 ± 2.7 ****	57.9 ± 3.5 ****
**PFOS 10 µM**	95.0 ± 1.1	92.7 ± 1.1 *	94.8 ± 1.4 **	**$CdCl_2$ 10 µM**	9.7 ± 2.3 ****	2.2 ± 0.9 ****	17.1 ± 3.6 ****
**72 h**	**Resazurin**	**Neutral red**	**CFDA-AM**	**72 h**	**Resazurin**	**Neutral red**	**CFDA-AM**
**Control**	100.0 ± 1.7	100.0 ± 2.3	100.0 ± 0.9	**Control**	100.0 ± 1.8	100.0 ± 0.6	100.0 ± 1.3
**PFOS 100 pM**	97.6 ± 2.3	100.9 ± 2.2	97.3 ± 1.2	**$CdCl_2$ 100 pM**	92.2 ± 2.0	95.9 ± 0.8 *	95.3 ± 1.2
**PFOS 1 nM**	95.6 ± 2.3	101.3 ± 2.2	96.2 ± 1.2	**$CdCl_2$ 1 nM**	88.5 ± 2.3 **	92.1 ± 0.8 ****	94.2 ± 1.5
**PFOS 10 nM**	94.2 ± 2.5	97.3 ± 2.4	96.8 ± 1.2	**$CdCl_2$ 10 nM**	89.5 ± 2.2 *	91.9 ± 0.8 ****	94.0 ± 1.4
**PFOS 100 nM**	97.3 ± 2.6	101.7 ± 2.8	96.6 ± 1.3	**$CdCl_2$ 100 nM**	91.1 ± 2.3	92.3 ± 1.2 ****	94.1 ± 1.5
**PFOS 1 µM**	97.3 ± 2.1	99.8 ± 2.3	96.8 ± 1.1	**$CdCl_2$ 1 µM**	86.9 ± 2.7 **	90.1 ± 1.4 ****	92.4 ± 1.6 **
**PFOS 10 µM**	102.3± 2.3	101.0 ± 2.8	98.5 ± 1.1	**$CdCl_2$ 10 µM**	0 ± 0.3 ****	53.2 ± 1.4 ****	10.8 ± 0.4 ****
**48 h**	**Resazurin**	**Neutral red**	**CFDA-AM**	**48 h**	**Resazurin**	**Neutral red**	**CFDA-AM**
**Control**	100.0 ± 1.6	100.0 ± 1.0	100.0 ± 1.0	**Control**	100.0 ± 0.9	100.0 ± 0.8	100.0 ± 1.0
**DEHP 100 pM**	95.8 ± 1.9	99.0 ± 1.4	96.7 ± 1.5	**DDE 100 pM**	95.6 ± 1.3	102.9 ± 1.5	97.0 ± 1.5
**DEHP 1 nM**	94.1 ± 2.0	97.8 ± 1.0	96.7 ± 1.3	**DDE 1 nM**	95.6 ± 1.5	100.9 ± 2.3	95.9 ± 1.2
**DEHP 10 nM**	92.9 ± 2.2	94.1 ± 1.3 **	95.5 ± 1.7	**DDE 10 nM**	85.4 ± 1.8 ****	89.1 ± 1.9 **	93.5 ± 1.6 **
**DEHP 100 nM**	94.1 ± 2.2	99.6 ± 1.4	96.0 ± 1.4	**DDE 100 nM**	94.4 ± 1.3 *	101.2 ± 1.8	99.9 ± 1.4
**DEHP 1 µM**	91.3 ± 2.5 *	93.5 ± 1.2 **	94.7 ± 1.4 *	**DDE 1 µM**	91.1 ± 1.4 ***	95.2 ± 1.5	96.1 ± 1.3
**DEHP 10 µM**	92.5 ± 3.5	79.5 ± 1.7 ****	83.2 ± 3.2 ****	**DDE 10 µM**	77.4 ± 4.1 ****	79.5 ± 4.5 ****	87.6 ± 2.2 ***
**72 h**	**Resazurin**	**Neutral red**	**CFDA-AM**	**72 h**	**Resazurin**	**Neutral red**	**CFDA-AM**
**Control**	100.0 ± 1.6	100.0 ± 1.7	100.0 ± 1.4	**Control**	100.0 ± 1.7	100.0 ± 1.0	100.0 ± 1.1
**DEHP 100 pM**	94.3 ± 1.9	96.2 ± 1.8	93.6 ± 0.9 **	**DDE 100 pM**	99.5 ± 3.7	111.4 ± 2.7 *	99.1 ± 1.6
**DEHP 1 nM**	95.4 ± 1.6	99.0 ± 2.0	95.3 ± 1.0 *	**DDE 1 nM**	94.4 ± 4.1	106.8 ± 2.8	96.6 ± 2.0
**DEHP 10 nM**	93.3 ± 1.9 *	96.8 ± 1.4	95.2 ± 1.0 *	**DDE 10 nM**	96.5 ± 4.7	113.5 ± 3.0 **	97.9 ± 2.3
**DEHP 100 nM**	95.3 ± 1.7	99.2 ± 2.1	96.8 ± 0.9	**DDE 100 nM**	94.5 ± 5.0	113.9 ± 3.2 **	96.9 ± 2.4
**DEHP 1 µM**	88.6 ± 2.2 ***	90.7 ± 1.9 **	94.6 ± 1.0 *	**DDE 1 µM**	93.5 ± 4.5	109.1 ± 2.6	95.1 ± 2.1
**DEHP 10 µM**	59.1 ± 3.7 ****	47.1 ± 2.9 ****	65.9 ± 3.3 ****	**DDE 10 µM**	40.7 ± 2.4 *	82.4 ± 6.2	69.9 ± 5.1 *

Supplemental Table S1. Viability of MIN6 cells treated for 48 or 72 h with different BPA, BPS, $CdCl_2$, PFOS, DDE or DEHP concentrations (100 pM–10 µM) as evaluated by RZ, NR and CFDA-AM assays. n= at least three independent experiments. * vs. Control; *$p < 0.05$, **$p < 0.01$, ***$p < 0.001$ and ****$p < 0.0001$, one-way ANOVA followed by Dunnet's post hoc test or Kruskal-Wallis followed by Dunn's post hoc test. All data are expressed as mean ± SEM.

EndoC-βH1							
**24 h**	**Resazurin**	**Neutral red**	**CFDA-AM**	**24 h**	**Resazurin**	**Neutral red**	**CFDA-AM**
**Control**	100.0 ± 0.5	100.0 ± 2.0	100.0 ± 0.8	**Control**	100.0 ± 1.0	100.0 ± 1.2	100.0 ± 0.7
**BPA 1 nM**	100.0 ± 1.5	103.5 ± 1.8	98.3 ± 2.0	**BPS 1 nM**	103.8 ± 1.4	104.4 ± 1.8	102.8 ± 1.3
**BPA 10 nM**	99.1 ± 1.4	104.3 ± 1.5	99.0 ± 1.3	**BPS 10 nM**	105.4 ± 1.2 *	107.5 ± 2.0 *	104.4 ± 1.3
**BPA 100 nM**	98.3 ± 1.3	104.2 ± 2.2	97.9 ± 1.1	**BPS 100 nM**	102.7 ± 1.6	101.3 ± 3.2	104.7 ± 2.4
**BPA 1 μM**	96.2 ± 1.4	103.5 ± 2.8	96.2 ± 1.3	**BPS 1 μM**	95.9 ± 1.9	98.4 ± 1.2	95.7 ± 1.6
**48 h**	**Resazurin**	**Neutral red**	**CFDA-AM**	**72 h**	**Resazurin**	**Neutral red**	**CFDA-AM**
**Control**	100.0 ± 0.9	100.0 ± 1.8	100.0 ± 1.2	**Control**	100.0 ± 0.9	100.0 ± 1.5	100.0 ± 0.7
**BPA 1 nM**	100.4 ± 0.5	106.9 ± 1.2	99.3 ± 0.8	**BPS 1 nM**	100.7 ± 1.5	105.0 ± 2.5	98.7 ± 2.1
**BPA 10 nM**	98.3 ± 1.0	105.6 ± 2.0	97.1 ± 1.1	**BPS 10 nM**	99.2 ± 2.3	103.3 ± 2.4	99.4 ± 1.9
**BPA 100 nM**	96.1 ± 1.4	104.6 ± 2.3	94.7 ± 1.5 *	**BPS 100 nM**	104.3 ± 1.8	110.9 ± 2.9 **	100.4 ± 2.5
**BPA 1 μM**	94.9 ± 1.5 **	98.1 ± 3.4	93.1 ± 1.5 ***	**BPS 1 μM**	97.5 ± 1.8	97.0 ± 1.5	95.1 ± 1.2
**24 h**	**Resazurin**	**Neutral red**	**CFDA-AM**	**24 h**	**Resazurin**	**Neutral red**	**CFDA-AM**
**Control**	100.0 ± 0.4	100.0 ± 1.5	100.0 ± 0.8	**Control**	100.0 ± 0.8	100.0 ± 0.8	100.0 ± 1.3
**PFOS 1 nM**	99.4 ± 0.7	104.6 ± 1.3*	99.0 ± 0.9	**$CdCl_2$ 1 nM**	99.7 ± 1.3	101.2 ± 1.4	99.6 ± 1.7
**PFOS 10 nM**	98.0 ± 0.8	108.5 ± 1.1****	95.4 ± 1.2	**$CdCl_2$ 10 nM**	98.5 ± 1.5	102.3 ± 1.8	102.5 ± 1.6
**PFOS 100 nM**	97.4 ± 0.6 *	106.8 ± 0.9 **	94.4 ± 1.4 *	**$CdCl_2$ 100 nM**	98.5 ± 1.5	101.7 ± 1.7	103.9 ± 2.2
**PFOS 1 μM**	98.3 ± 0.8	107.3 ± 1.5 ***	92.8 ± 2.1 ***	**$CdCl_2$ 1 μM**	97.2 ± 0.9	97.7 ± 1.7	102.7 ± 3.1
**48 h**	**Resazurin**	**Neutral red**	**CFDA-AM**	**48 h**	**Resazurin**	**Neutral red**	**CFDA-AM**
**Control**	100.0 ± 0.3	100.0 ± 0.9	100.0 ± 0.9	**Control**	100.0 ± 0.4	100.0 ± 0.5	100.0 ± 1.0
**PFOS 1 nM**	98.7 ± 0.5	107.5 ± 0.7 ****	102.2 ± 1.7	**$CdCl_2$ 1 nM**	95.6 ± 0.8 **	101.2 ± 1.0	101.1 ± 1.1
**PFOS 10 nM**	96.8 ± 0.8 **	111.3 ± 1.1 ****	98.8 ± 1.7	**$CdCl_2$ 10 nM**	93.0 ± 1.2 ****	102.5 ± 1.4	103.1 ± 1.6
**PFOS 100 nM**	98.1 ± 0.9	105.9 ± 1.2 ***	96.9 ± 2.0	**$CdCl_2$ 100 nM**	92.1 ± 1.2 ****	102.7 ± 1.8	101.6 ± 1.8
**PFOS 1 μM**	99.3 ± 0.7	107.0 ± 1.5 ****	92.8 ± 2.1	**$CdCl_2$ 1 μM**	96.3 ± 0.7 *	99.7 ± 1.5	103.0 ± 2.5
**24 h**	**Resazurin**	**Neutral red**	**CFDA-AM**	**24 h**	**Resazurin**	**Neutral red**	**CFDA-AM**
**Control**	100.0 ± 0.3	100.0 ± 1.0	100.0 ± 1.4	**Control**	100.0 ± 0.6	100.0 ± 0.9	100.0 ± 1.0
**DEHP 1 nM**	98.8 ± 0.6	103.4 ± 1.5	100.8 ± 1.9	**DDE 1 nM**	98.4 ± 0.7	100.8 ± 1.2	99.4 ± 0.7
**DEHP 10 nM**	98.3 ± 0.7	103.9 ± 1.1	101.7 ± 1.7	**DDE 10 nM**	97.5 ± 0.8	101.9 ± 1.2	97.4 ± 0.8
**DEHP 100 nM**	97.0 ± 0.8 *	102.5 ± 1.2	98.2 ± 1.5	**DDE 100 nM**	95.5 ± 0.7 ***	99.7 ± 1.1	95.4 ± 1.2 **
**DEHP 1 μM**	98.6 ± 1.2	100.1 ± 2.1	93.5 ± 2.2*	**DDE 1 μM**	94.5 ± 1.2 ****	96.9 ± 1.1	95.7 ± 0.9 **
**48 h**	**Resazurin**	**Neutral red**	**CFDA-AM**	**48 h**	**Resazurin**	**Neutral red**	**CFDA-AM**
**Control**	100.0 ± 0.4	100.0 ± 0.8	100.0 ± 1.5	**Control**	100.0 ± 0.6	100.0 ± 0.4	100.0 ± 0.9
**DEHP 1 nM**	100.1 ± 1.1	102.9 ± 0.7	98.3 ± 2.3	**DDE 1 nM**	100.7 ± 0.9	104.8 ± 0.9 ***	100.6 ± 1.0
**DEHP 10 nM**	99.3 ± 0.9	107.4 ± 1.8 *	106.7 ± 2.8	**DDE 10 nM**	96.8 ± 1.6	106.2 ± 0.6 ****	95.5 ± 1.6
**DEHP 100 nM**	97.5 ± 0.7	106.9 ± 1.6 *	98.3 ± 1.1	**DDE 100 nM**	96.7 ± 1.4	104.6 ± 0.7 ***	97.6 ± 1.8
**DEHP 1 μM**	101.7 ± 0.9	100.1 ± 2.8	85.9 ± 2.4 ****	**DDE 1 μM**	96.5 ± 0.9 *	95.3 ± 1.2 ***	94.8 ± 1.5 *
**72 h**	**Resazurin**	**Neutral red**	**CFDA-AM**	**72 h**	**Resazurin**	**Neutral red**	**CFDA-AM**
**Control**	100.0 ± 0.6	100.0 ± 1.5	100.0 ± 1.0	**Control**	100.0 ± 1.1	100.0 ± 1.4	100.0 ± 1.0
**DEHP 1 nM**	95.3 ± 1.2 *	102.5 ± 1.9	91.7 ± 1.6 **	**DDE 1 nM**	98.9 ± 1.7	100.6 ± 2.5	100.0 ± 1.9
**DEHP 10 nM**	99.5 ± 0.8	104.8 ± 2.1	100.3 ± 2.5	**DDE 10 nM**	99.4 ± 1.8	103.6 ± 2.5	100.0 ± 1.8
**DEHP 100 nM**	98.5 ± 0.6	105.6 ± 2.1	98.1 ± 2.5	**DDE 100 nM**	100.3 ± 1.1	103.2 ± 2.0	100.7 ± 1.7
**DEHP 1 μM**	99.4 ± 1.4	98.3 ± 3.2	85.4 ± 3.0 ***	**DDE 1 μM**	97.4 ± 0.8	96.7 ± 1.6	96.5 ± 1.5

Supplemental Table S2. Viability of EndoC-βH1 cells treated for 24, 48 or 72 h with different BPA, BPS, $CdCl_2$, PFOS, DDE or DEHP concentrations (1 nM–1 μM) as evaluated by RZ, NR and CFDA-AM assays. n= at least four independent experiments. * vs. Control; *p < 0.05, **p < 0.01, ***p < 0.001 and ****p < 0.0001, one-way ANOVA followed by Dunnet's post hoc test or Kruskal-Wallis followed by Dunn's post hoc test. All data are expressed as mean ± SEM.

	GEN	FORWARD (5'->3')	REVERSE (5'->3')	REFSEQ (NM)
MIN6	*Ins*	TTATTGTTTCAACATGGCCC	CAAAGGTGCTGCTTGACAAA	001185083
	Pdx1	GGCCTGGAAGAGCCCAACCG	TGTGTAAGCACCTCCTGCCCACT	008814
	Hnf4α	TCTGGATGACCAGGTGGCGCT	GGACACACGGCTCATCTCCGC	008261
	MafA	CCCGTCTTGGCCCTCCATGATT	TATCCCGCCGTGTCTGTGTGG	194350
	Kir6.2	CCCGTCTTGGCCCTCCATGATT	TATCCCGCCGTGTCTGTGTGG	010602
	Sur1	TGCCTCAGGACAAGCAACC	GACCACTGTCCTCTTGTCATC	011510
	Glut2	ATCGCTCCAACCACACTCAG	CTGAGGCCAGCAATCTGACTA	031197
	Gck	TTCAGCTTCTGGCCTCCCACAG	AAAACAGCCAGGTCTGGGCAGC	010292
	Hprt	GGTTAAGCAGTACAGCCCCA	TCCAACACTTCGAGAGGTCC	013556
	Actb	GGCTGTATTCCCCTCCATCG	CCAGTTGGTAACAATGCCATGT	007393
EndoC-βH1	*INS*	AAGCAGATCACTGTCCTTC	ACACTAGGTAGAGAGCTTCC	000207
	PDX1	CCTTTCCCATGGATGAAGTC	CGAACTCCTTCTCCAGCTCTA	000209
	HNF4α	CGAAGGTCAAGCTATGAGGACA	ATCTGCGATGCTGGCAATCT	178849
	MAFA	TTCTCCTTGTACAGGTCCCG	GAGAGCGAGAAGTGCCAACT	201589
	MAFB	AAGCCTCTCACCCTAGGAGC	GGAAAACAGATCCTCCCCTC	005461
	KIR6.2	AGGTCCAAGTGACTATTGGCT	TCTGCACGATGAGGATCAGGA	000525
	SUR1	TCGTGAATCTGCTGTCCAAAG	CGATGGCTCGCAAGTCGAT	001287174
	SNAP25	TCGTGTAGTGGACGAACGG	TCTCATTGCCCATATCCAGGG	003081
	GLUT1	TCTGGCATCAACGCTGTCTTC	CGATACCGGAGCCAATGGT	006516
	GCK	TGCTACTACGAAGACCATCAGT	CCACTCGGTATTGACGCACA	000162
	HPRT1	GCCCTGGCGTCGTGATTAGT	AGCAAGACGTTCAGTCCTGTCCATAA	000194
	ACTB	CACCATTGGCAATGAGCGGTTC	AGGTCTTTGCGGATGTCCACGT	001101

Supplemental Table S3. Primer sequences used in RT-qPCR for the study of pancreatic β-cell gene expression.
